# Functionalized Fluorescent Nanodiamonds for Simultaneous Drug Delivery and Quantum Sensing in HeLa Cells

**DOI:** 10.1021/acsami.2c11688

**Published:** 2022-08-19

**Authors:** Yuchen Tian, Anggrek C. Nusantara, Thamir Hamoh, Aldona Mzyk, Xiaobo Tian, Felipe Perona Martinez, Runrun Li, Hjalmar P. Permentier, Romana Schirhagl

**Affiliations:** † Department of Biomedical Engineering, Groningen University, University Medical Center Groningen, Antonius Deusinglaan 1, 9713 AW Groningen, Netherlands; ‡ Department of Analytical Biochemistry, Interfaculty Mass Spectrometry Center, Groningen Research Institute of Pharmacy, University of Groningen, A. Deusinglaan 1, Groningen 9713 AV, The Netherlands; ⊥ Institute of Metallurgy and Materials Science Polish Academy of Sciences, 25 Reymonta Street, 30-059, Cracow, Poland

**Keywords:** NV center, fluorescent biomarker, free radical, nanodiamonds, quantum sensing, intracellular sensors, drug delivery

## Abstract

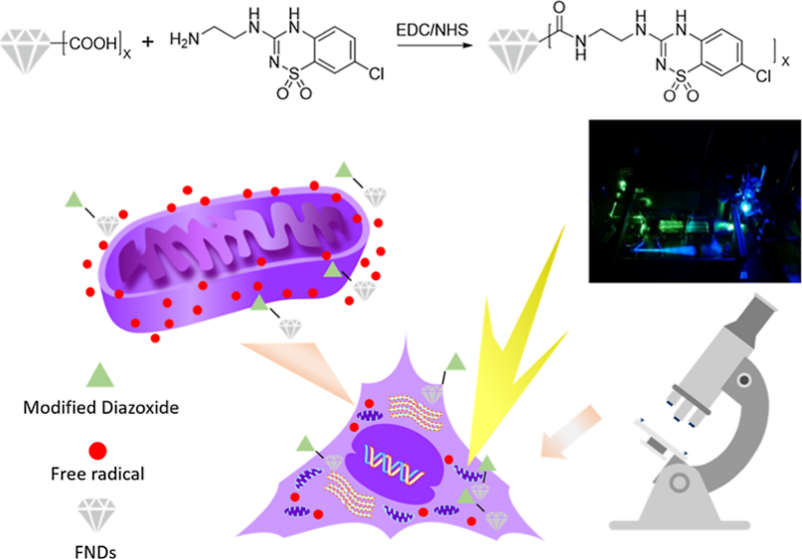

Here, we present multifunctional fluorescent nanodiamonds (FNDs) for simultaneous drug delivery and free radical detection. For this purpose, we modified FNDs containing nitrogen vacancy (NV) centers with a diazoxide derivative. We found that our particles enter cells more easily and are able to deliver this cancer drug into HeLa cells. The particles were characterized by infrared spectroscopy, dynamic light scattering, and secondary electron microscopy. Compared to the free drug, we observe a sustained release over 72 h rather than 12 h for the free drug. Apart from releasing the drug, with these particles, we can measure the drug’s effect on free radical generation directly. This has the advantage that the response is measured locally, where the drug is released. These FNDs change their optical properties based on their magnetic surrounding. More specifically, we make use of a technique called relaxometry to detect spin noise from the free radical at the nanoscale with subcellular resolution. We further compared the results from our new technique with a conventional fluorescence assay for the detection of reactive oxygen species. This provides a new method to investigate the relationship between drug release and the response by the cell via radical formation or inhibition.

## 1 Introduction

In the last few years, fluorescent nanodiamonds (FNDs) have become increasingly popular. They are excellently suited for drug delivery for several reasons. They are biocompatible, which has been demonstrated in many different cell types and animal models.^1,2^ They are readily ingested via the natural endocytosis pathways by many different cells types, including many cancer cells.^3−5^ Additionally, FNDs have an inert core but a rich surface chemistry, which can be utilized to attach many different drugs.^6,7^ This has already been demonstrated for several different drugs, including, for instance, cancer drugs,^8,9^ siRNA,^10^ drugs for HIV,^11^ insulin^12^ (as used by diabetics), or therapeutic antibodies.^13^ Further useful is their relatively small size.^14,15^ Since FNDs are uniquely photostable and do not bleach or blink, they can be observed via their fluorescence, which can be useful for biodistribution studies.^16^

Apart from drug delivery applications, FNDs have also drawn attention because of their unique optical properties.^17−19^ Since they do not bleach, FNDs are especially useful for long-term optical imaging.^20^ It is also worth mentioning that FNDs are well visible with many different imaging techniques and are thus attractive labels for correlative microscopy.^21,22^

Another remarkable property is their ability for magnetic sensing. FNDs contain defects called nitrogen vacancy (NV) centers, which sense magnetic resonances optically by changing their fluorescence based on the magnetic surrounding. They are so sensitive that they can even detect a single electron or even a few nuclear spins.^23,24^ This method has already been applied in several applications including the measurements of magnetic nanoparticles, magnetic domain walls,^33^ or the presence of molecules on the diamond surface.^25−27^ Also, a few biological applications have been demonstrated including temperature sensing in cells,^28^ measurements of spin labels,^29^ iron-containing proteins,^30^ or magnetic particles.^31^ Last year, we demonstrated free radical detection in cells.^32−34^

Free radicals in cells can lead to damage to nucleic acids, proteins, and lipids. Radical-related damage plays a vital role in multiple diseases, such as cancer,^35^ bacterial or viral infection or cardiovascular diseases. For this reason, there is interest in tracking free radicals to monitor the health status of cells.^36−38^ A common method to measure free radicals is using fluorescent probes, which react with free radicals to form fluorescent compounds.^39^ However, because of bleaching of these fluorescent compounds, this method does not allow long term observation. Additionally, the chemical reaction leading to a fluorescent compound is irreversible. Thus, this method can only measure the history of a sample while we provide the current stage.^39^

While both drug delivery and quantum sensing of free radicals have been achieved with FNDs individually, we combine the two methods for the first time. This has the advantage that one can deliver a drug and observe the response of the delivered drug directly. We developed FND–diazoxide complexes and used them to deliver the adsorbed drug. Diazoxide influences K^+^ channels in mitochondria, which affect the release of free radicals and increase the insulin level to suppress cancer growth.^40−42^ Without losing its function, it is also possible to alter chemical groups in diazoxide, which allow linking the molecule to particles or other molecules.^43−46^ In our case, aminated diazoxide was used as a ligand, which can react with active carboxyl groups on the surface of FNDs. When the composite system enters the cell, we can locate the particles in real-time and detect changes in free radical concentrations inside cells by FNDs.

## 2 Materials and Methods

### 2.1 Materials

**Figure 1:**
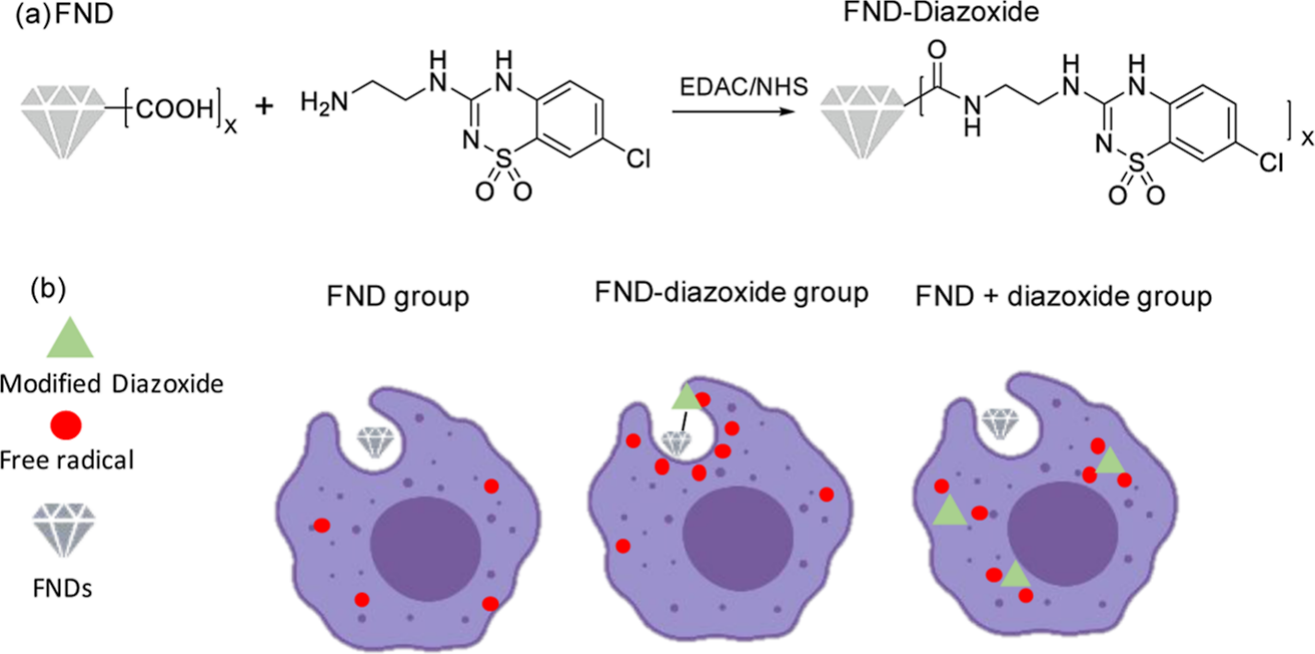
Experimental design. (a) Shows the chemical reaction that is performed to obtain FND–diazoxide. (b) Summarizes the different experimental groups that are compared in this article: the FND group using bare particles, the FND–diazoxide group where a diazoxide derivative is linked covalently to the diamond surface, and the FND + diazoxide group where both FNDs and Diazoxide are supplied separately.

FNDs were obtained from Adámas Nano (Raleigh, NC, USA). These particles are produced by the vendor via HPHT synthesis followed by grinding. According to the vendor, the particles are irradiated with 3MeV electrons. The irradiation was performed using a fluence of 5*10^19^ e/cm^2^. As a result, these particles contain 500 NV centers per diamond on average (determined by EPR by the manufacturer^47^). After high-temperature (600 °C) annealing, the material is cleaned with oxidizing acids and is thus oxygen-terminated. The material is widely used and thus characterized well in the literature already.^48,49^ 1,2-Ethanediamine and N1-(7-chloro-1,1-dioxido-2*H*-1,2,4-benzothiadiazin-3-yl) (a diazoxide derivative with an amine group, shown in Figure 1) was purchased from Chemhere Co. Ltd. (Hong Kong, China). 2,3-Bis-(2-methoxy-4-nitro-5-sulfophenyl)-2*H*-tetrazolium-5-carboxanilide (XTT), 1-ethyl-3-(3-dimethylaminopropyl) carbodiimide hydrochloride (EDAC), and *N*-hydroxysuccinimide (NHS) were purchased from Sigma-Aldrich (USA). Dulbecco’s Modified Eagle Medium (DMEM), fetal bovine serum (FBS), and penicillin/streptomycin were purchased from Gibco (The Netherlands).

### 2.2 Conjugation of Diazoxide to FNDs

Since there are carboxyl groups on the surface of FNDs,^49^ and 1,2-ethanediamine, N1-(7-chloro-1,1-dioxido-2*H*-1,2,4-benzothiadiazin-3-yl) (modified diazoxide) has an amine group, it is possible to conjugate them via an EDAC/NHS activation method.^50^ The reaction is shown in Figure 1. Briefly, an ice-cold mixture of 1.0 mL of EDAC (25 μg/mL in water) and 1.0 mL of NHS (15 μg/mL in water) was added into 2.0 mL of the FND solution (50 μg/mL in water) under continuous magnetic stirring for 20 min. Then 2.0 mL of the amino diazoxide solution (25 μg/mL in water) was added dropwise and left at room temperature for 16 h. The FND–diazoxide solution was ultracentrifuged (3000 g, 45 min), and the filtrate was stored for further analysis. Unreacted modified diazoxide in the filtrate was quantified with high-pressure liquid chromatography–ultraviolet (HPLC–UV).^51^ To this end, unreacted modified diazoxide was lyophilized and dissolved in 1 mL of acetonitrile. In this solution, the molecule was quantified using HPLC (Shimadzu, Kyoto, Japan). The HPLC system was equipped with a SIL-20AC autosampler and two LC-20AT pumps. To detect the absorbance, an SPD-20A detector was used. Diazoxide (30 μL was injected each time) was separated on a Vydac RP-C18 column (250 mm × 4.6 mm i.d., 5 μm particles, 300 Å pore size, Grace Vydac, Lokeren, Belgium). For the separation, we used a 30 min gradient of 2–30% acetonitrile in water/0.1% formic acid at a flow rate of 1 mL/min. Elution of diazoxide was detected at 254 nm. The peak area of the supernatant was converted into a concentration by using a calibration curve constructed with standard diazoxide solutions. We then used a mass balance to calculate the amount of diazoxide that was attached to the surface of FNDs.

### 2.3 Characterization

Transmission electron microscopy (TEM) images were measured using a Tecnai T20 electron microscope (FEI, The Netherlands), operating at 200 keV. Samples were deposited on a plain carbon film on 400 mesh copper TEM grids. Images were recorded on a slow scan CCD camera to visualize the structure and morphology of FNDs and FND-diazoxide.

We used a size zeta potential analyzer (Nano-ZS, Malvern, England) to determine the particle size and zeta potential of the FND-diazoxide and bare FNDs at room temperature. The results were expressed as averages of the mean diameter obtained from three measurements.

The functional groups present in FNDs before and after modification were investigated by a Fourier-transform infrared spectrometer (Cary 670, Agilent Technologies, USA).

### 2.4 Cell Culture

HeLa cells, cells from a human cervical cancer cell line, were cultured in DMEM. This medium was supplemented with 10 wt % FBS, 100 units/mL penicillin, and 100 mg/mL streptomycin. The cells were cultured in an incubator at 37 °C and 5% CO_2_.

### 2.5 Diamond Uptake

We defined and quantified ingested diamonds by laser confocal microscopy using a 780 system (Zeiss, Sliedrecht, The Netherlands). 2 mL of HeLa cells in DMEM was seeded into 35 mm glass-bottom dishes (Greiner Bio-One, Austria) and cultured overnight. On the following day, the medium was replaced by fresh DMEM. Depending on the experimental group, the medium either contained 5 μg/mL FNDs, 5 μg/mL FND–diazoxide, 5 μg/mL the mixture of FNDs and modified diazoxide (weight ratio, 10:1), or PBS buffer. After 12 h of further incubation, the cells were washed with 1 mL of PBS to remove excess diamonds that were not taken up.

For LSCM imaging, the cells were fixed in 3.7% PFA (paraformaldehyde) for 30 min and then washed three times with 1 mL of PBS buffer. Then, the HeLa cells were treated with 0.5% Triton for 5 min and blocked in 5% PBSA (bovine serum albumin in phosphate-buffered saline). Then, we stained the cells using 2 μg/mL phalloidin-FITC (Sigma-Aldrich, Zwijndrecht, The Netherlands) to label f-actin of cytoskeleton in 1% PBSA and 2 μg/mL DAPI (Sigma-Aldrich, Zwijndrecht, The Netherlands) to label the nuclei. The samples were excited at 405 nm for nuclei, 488 nm for cytoskeleton, and 561 nm for FNDs. The emission wavelengths were 424–485, 499–552, and 645–758 nm, and images were analyzed using FIJI 2.0. software (https://fiji.sc).

### 2.6 Cell Viability Assays

Cytotoxicity of FNDs to the HeLa cells was measured by the XTT method. HeLa cells at a concentration of 1 × 10^4^ cells/well were seeded in DEMEM into microplates (tissue culture grade, 96 wells, flat bottom). After 24 h incubation, the medium was replaced with fresh DMEM with different concentrations of FNDs, FND–diazoxide, or the mixture of FNDs and the modified diazoxide (weight ratio, 10:1). Then, cells were cultured for another 24 h at 37 °C and 5% CO_2_ in an incubator. Then, 50 μL of the XTT labeling mixture was added to each well, and the mixture was incubated for 3 h. Next, HeLa cells were dissolved in 2-propanol, resulting in a purple solution. The absorption of this solution was measured at 690 nm using a FLUOstar Omega microplate reader (BMG Labtech, De Meern, The Netherlands). After correction and comparison to the background, this indicates metabolic activity and thus the viability of the cells.

### 2.7 Microscopic Analysis

Then, we observed morphological changes to assess any potential effects of the diamond uptake on the cytoskeleton. Then, a homemade FND quantification plugin was used to estimate the amount of internalized FNDs. The analysis that was already described elsewhere^52,53^ was performed in three phases: cell selection, masking, and particle analysis. First, our program selected cells randomly for the analysis. Cells with large diamond aggregates on the cell membrane were rejected since large aggregates lead to false positives also in close slices. The cell volume was defined in 3D within z stacks. In the z-dimension, we identified the first and last slices containing the cell. Then, the entire volume is molded in order to resemble the shape of the cell. This process is called the masking phase. The phalloidin-FITC signal was converted to binary utilizing the Isodata algorithm. To find the inner volume of each HeLa cell, the program removes the area that is closest to the membrane to exclude particles on the membrane from the analysis. Finally, we use a function of Fiji, which counts the objects (connected positive pixels) found in a selected region. Here, we call the amount of adjacent FND positive pixels an object. This means that an object can be either a single particle or an aggregate. Then, we use a threshold to determine if a specific pixel is an FND or background light. We assume that pixels with an intensity below the threshold are background (set as black), while pixels greater than or equal to the threshold are part of an object. As a threshold, we chose the lowest value, where we still obtained zero for a negative control image. In addition to the number of objects, we also determined the number of particles. To find the number of particles, we divide it by the number of pixels that form a single particle (determined from a comparison with a sample where particles are separated on a surface). Comparing the particle number and the object number reveals the aggregation behavior (in a sample with no aggregation, the number of objects and the number of particles would be the same).^54^

### 2.8 Total ROS Activity

We used 2′,7′-dichlorodihydrofluorescein diacetate (DCFDA) to determine the reactive oxygen species (ROS) production inside cells. After incubating with the cell for 3 or 12 h, DCFDA is deacetylated and, in the presence of ROS, oxidized to 2′,7′-dichlorodihydrofluorescein (DCF). The presence of DCF was detected via its green fluorescence (measured by a FLUOstar Omega microplate reader, excitation 485 nm and emission 520 nm). To perform the assay, we first added DCFDA (20 μM) in phenol red-free DMEM medium to the cells. Then, we incubated for 45 min before adding FNDs, FND–diazoxide, or the mixture of FNDs and modified diazoxide (weight ratio, 10:1), respectively. 50 μM *tert*-butyl hydroperoxide (TBHP) in the absence of FNDs was used as a positive control. Incubation with PBS was used as a negative control. For all samples, we subtracted the background from the medium without cells and related the fluorescence to the negative control. All measurements were performed in quadruplicate on independent samples.

### 2.9 Free Radical Detection

After the FND was found in the sample, its location inside the cell was confirmed using Z-stack imaging. Then, the free radical concentration was determined via relaxometry (also called T1).

**Figure 2:**
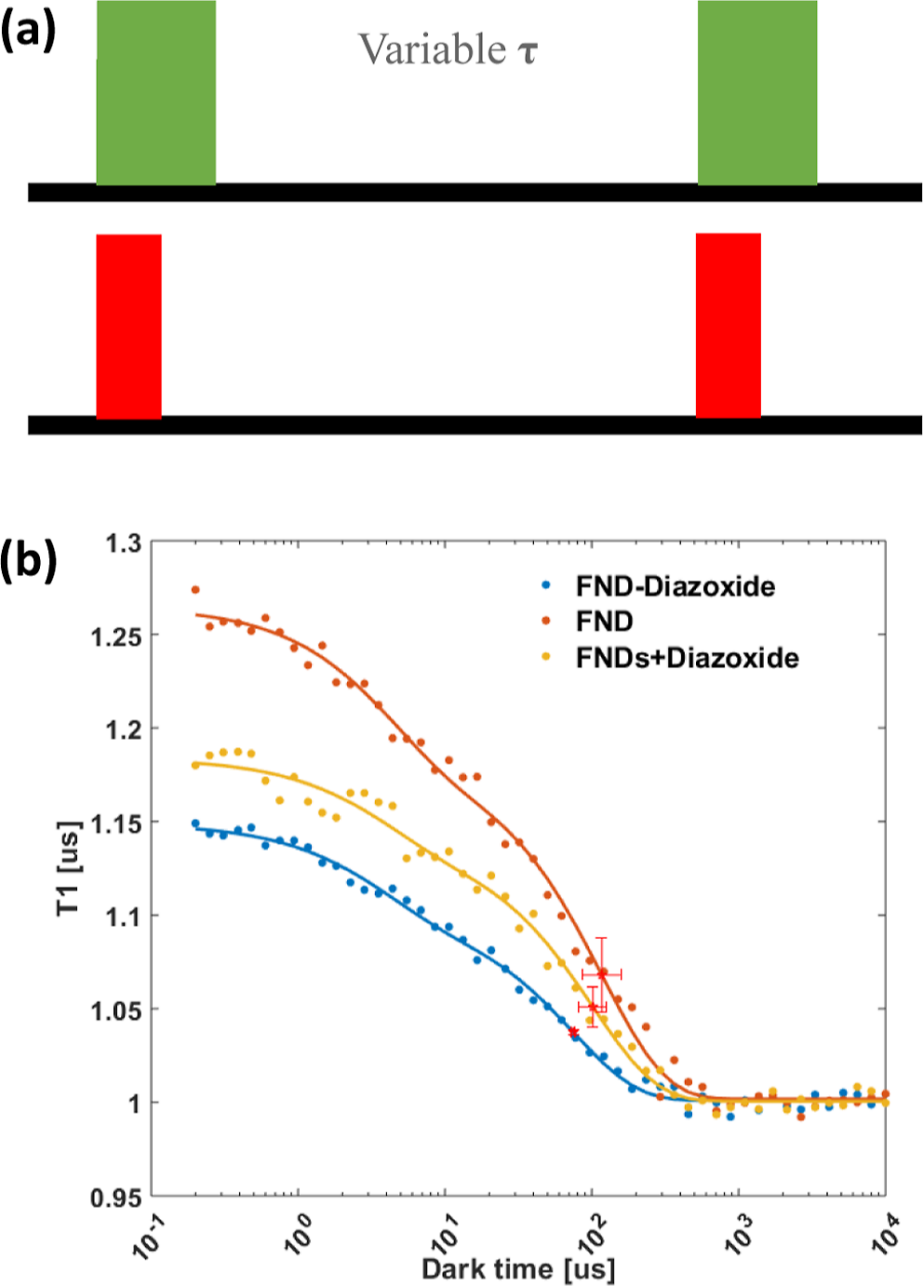
Performing T1 measurements. The T1 or relaxometry sequence we used for radical detection is shown in (a). Green bars represent time periods when the laser is on. In between, the laser is off for a varying dark period. At the beginning of each pulse, the fluorescence intensity is detected above 600 nm (indicated by red bars). If the intensities are plotted against different dark periods, as shown in (b), a decay curve is obtained. The faster the process, the higher the local radical load. The curves shown here were recorded from particles in HeLa cells.

T1 experiments were carried out using a home-built diamond magnetometer. Such a magnetometer is equipped with optics, which allows pulsing, and an avalanche photodiode (Excelitas, SPCM-AQRH) as the detector.^55^ To enable the pulsing, an acousto-optical modulator (Gooch & Housego, model 3350-199) was used. Using this technique, the magnetic noise from the material surrounding the FNDs was recorded by optical means. The NV centers were irradiated with a 532 nm laser for 5 μs (enough to achieve polarization of the NV centers) after dark times between 0.2 μs and 10 ms. The pulsing sequence (shown in Figure 2a) was repeated 10,000 times for each measurement to obtain a sufficient signal-to-noise ratio (the entire sequence took around 10 min). During each pulse, the NV centers were pumped into the (bright) ms = 0 state from the (dark) equilibrium between ms = 0 and ms = + or −1. The time this process takes is called the T1 time (relaxation time). This T1 time can be utilized to quantify the radical concentration in the FND’s surroundings. Representative curves are shown in Figure 2b. An oil objective (Olympus, UPLSAPO) with 100× magnification was used for collecting the light. We used a laser power of 50 μW at the location of the sample. This laser power is low enough to avoid damage to the cells but also high enough to polarize the NV centers. Our optical signal is equivalent to T1 in conventional magnetic resonance imaging but from nanoscale voxels. A tracking algorithm was used during the measurement since diamond particles move inside the cell.

The model we used to fit the data and calculate the relaxation time is described in eq 1.
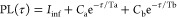
1T1 = max (Ta, Tb).

This model is not the same as the one that is mostly used for single NV centers^56^ because ensembles were used. The relaxation of the ensemble is approximated from two components^54^ from the NV centers with a short T1 (closer to the surface (Ta) or close to a source of noise within the crystal) and a long one [from less perturbed NV centers (Tb)].

The longer T1 time was selected between these two relaxation constants for analysis and quantification because we found that it is more sensitive to changes.^54^ The measurements were performed for known concentrations of diazoxide.

### 2.10 Drug Release Profile

In vitro drug release was determined using a dynamic dialysis method^58^ conducted at 37 °C. Typically, 0.5 mL of FND–diazoxide in PBS (pH 7.4) was inserted into a dialysis bag (MWCO: 3.5 KDa) and dialyzed against 25 mL of PBS (pH 7.4) in a water bath at 37 °C. At two-hour intervals, 0.5 mL of the sample was removed from the release medium, and an equal volume of PBS was added. The amount of diazoxide was determined using a Shimadzu UV detector (Japan) at 254 nm, and the percentage of cumulative release was calculated as reported previously.^59^

## 3 Results and Discussion

### 3.1 Preparation and Characterization of Diazoxide-Modified FNDs

**Figure 3:**
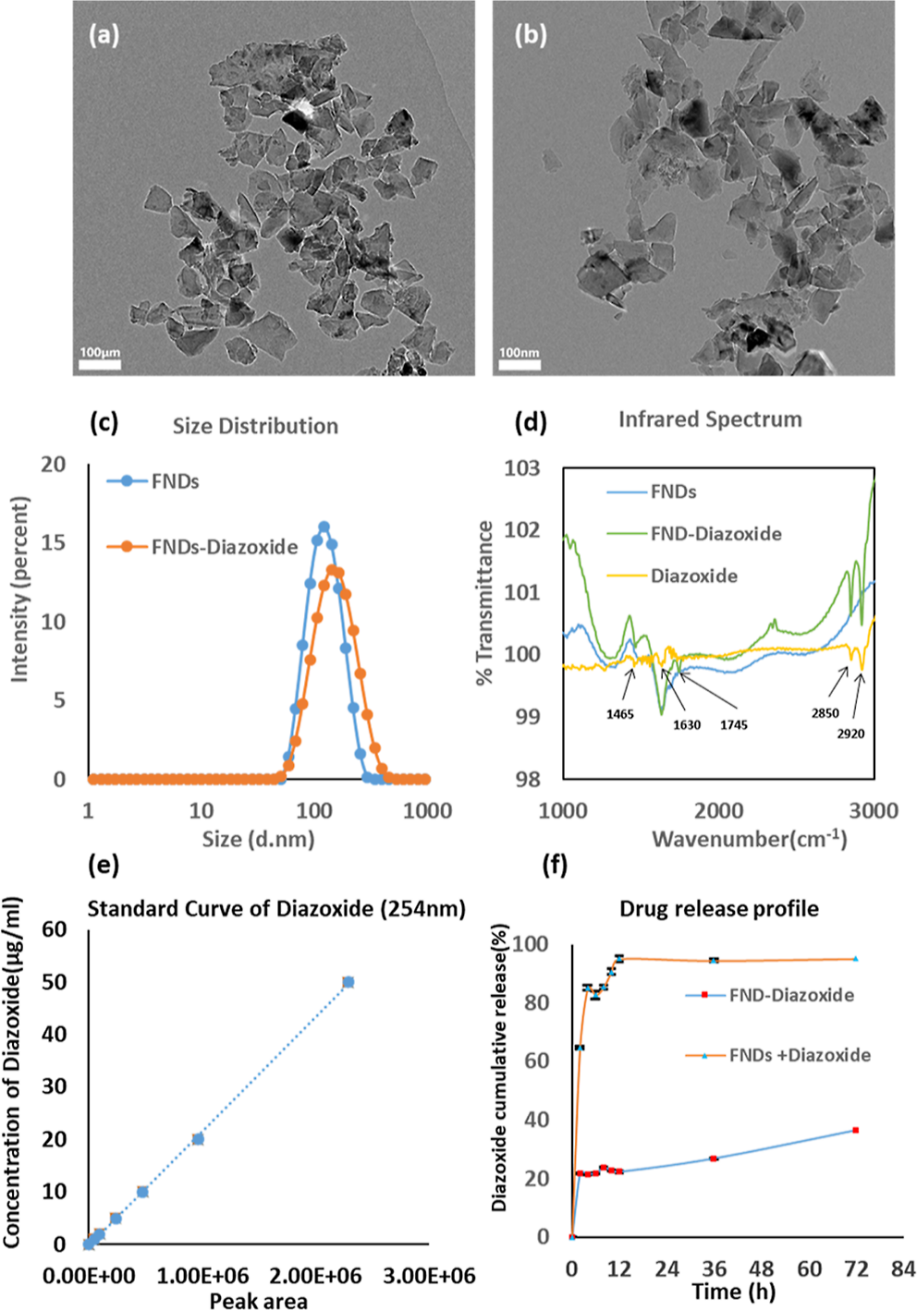
Characterization of FND–diazoxide. High-resolution TEM image of (a) FND–diazoxide and (b) FNDs. (c) Size distributions of FND–diazoxide and FNDs at room temperature measured by DLS at a scattering angle of 173° (backscatter detection). (d) Infrared spectrum of FND–diazoxide, FNDs, and diazoxide. (e) Standard curve of diazoxide determined by HPLC. (f) In vitro cumulative release of diazoxide from FND–diazoxide and the mixture of FNDs and diazoxide in PBS (pH = 7.4) for 72 h at 37 °C.

Carboxylic acid groups on the surface of FNDs were activated with EDAC and NHS and then reacted with amine groups of the modified diazoxide. In the infrared spectrum of diazoxide (Figure 3d), the peaks at 2920 and 2850 cm^–1^ are identified as a stretching vibration of imino groups, while the peak at 1630 cm^–1^ corresponds to the bending of amino groups. The peak at 1462 cm^–1^ is assigned to methyl and methylene groups. All these peaks also appeared in the infrared spectrum of FND–diazoxide (Figure 3d), except that a new peak appeared at 1745 cm^–1^, which is ascribed to carbonyl groups (Figure 3d). The appearance of the carbonyl group indicates that the carboxyl group on the surface of FNDs successfully reacted with the amino group from diazoxide. As the amount of modified diazoxide may affect free radical production in cells, the amount of modified diazoxide was checked after the conjugation experiment. After the reaction, unreacted modified diazoxide was determined by an HPLC-UV at a wavelength of 254 nm (Figure 3e). When we used 50 μg of modified diazoxide in the reaction with 100 μg FNDs, there was around 38.7 μg left. Thus, the weight ratio of diazoxide-to-FND in FND–Diazoxide is around 1:10. TEM, which indicates that the FND–diazoxide, as FNDs themselves, are irregular in shape and size. Dynamic light scattering (DLS) revealed a mean hydrodynamic size of 139.2 nm for FND–diazoxide, with a narrow size distribution (polydispersity index (PDI) < 0.191, Figure 3c). Compared to FNDs, for which the mean hydrodynamic diameter is 115.9 nm and the PDI is 0.098, the modification with diazoxide does not lead to obvious aggregation. This can be further understood by measuring the zeta potential of the particles. The zeta potentials before and after modification are −42.9 and −36.6 mV, respectively. These findings support that the coated particles remain stable.

### 3.2 Drug Release

Next, we followed the drug release characteristics in vitro. In Figure 3f, we show the cumulative drug release. We compared two groups: FNDs mixed with the drug and particles where the drug was attached covalently. While the drug is available quickly in the mixture, the covalently attached drug is released slowly from the particle surface. It is also worth noting that not 100% of the drug is released within 72 h when the drug was covalently attached. Owing to the strong binding, the release is much slower, and some drug molecules might even remain on the particles permanently. A similar behavior was also observed by Li et al,^59^ who reported between 30 and 40% release of doxorubicin from nanodiamonds within a similar time frame. Such sustained release is known for many different drug delivery nanoparticles.^60−62^ Similar release behavior from diamond particles was also already observed in the literature. Huang et al., Wang et al., and Li et al., for instance, described slow release of doxorubicin that was attached to nanodiamonds.^59,63,64^ While in this simplified case, the drug was released by hydrolysis of the amide bond that connects the drug molecule with the diamond surface, there are more factors that play a role in cancer in vivo. Apart from sustained release, FNDs have been reported to have a longer circulation time in the blood stream, and thus more of them might be able to end up in the tumors. Additionally, some tumors have the ability to excrete drug molecules, and nanodiamond loaded with drugs have been shown to escape this mechanism.^65^ Once in the cells, the cleaving of the amide bond is likely accelerated by the acidic environment in endosomes and later lysosomes, where nanodiamond particles have been reported to enter and reside in cells.^66−68^ Owing to these characteristics in the body, relatively low toxicity for the healthy cells has been reported for drug delivered with nanodiamonds.

### 3.3 Cellular Uptake of FNDs and FND–Diazoxide in HeLa Cells

**Figure 4:**
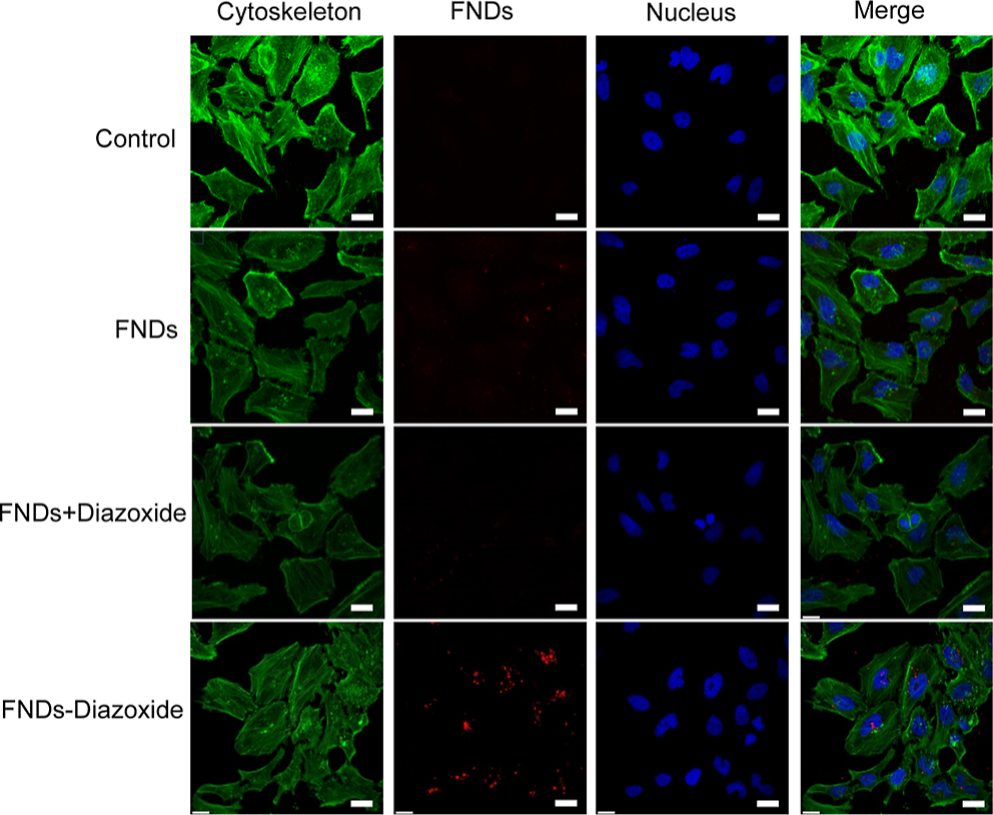
Nanodiamond uptake into HeLa cells. The control is a sample that did not contain nanodiamonds. The FND group contains bare nanodiamonds, while FND + diazoxide contains both diazoxide and bare nanodiamonds. Finally, the FND–diazoxide group contains FNDs linked to diazoxide. The scale bars are 25 μm in size.

**Figure 5:**
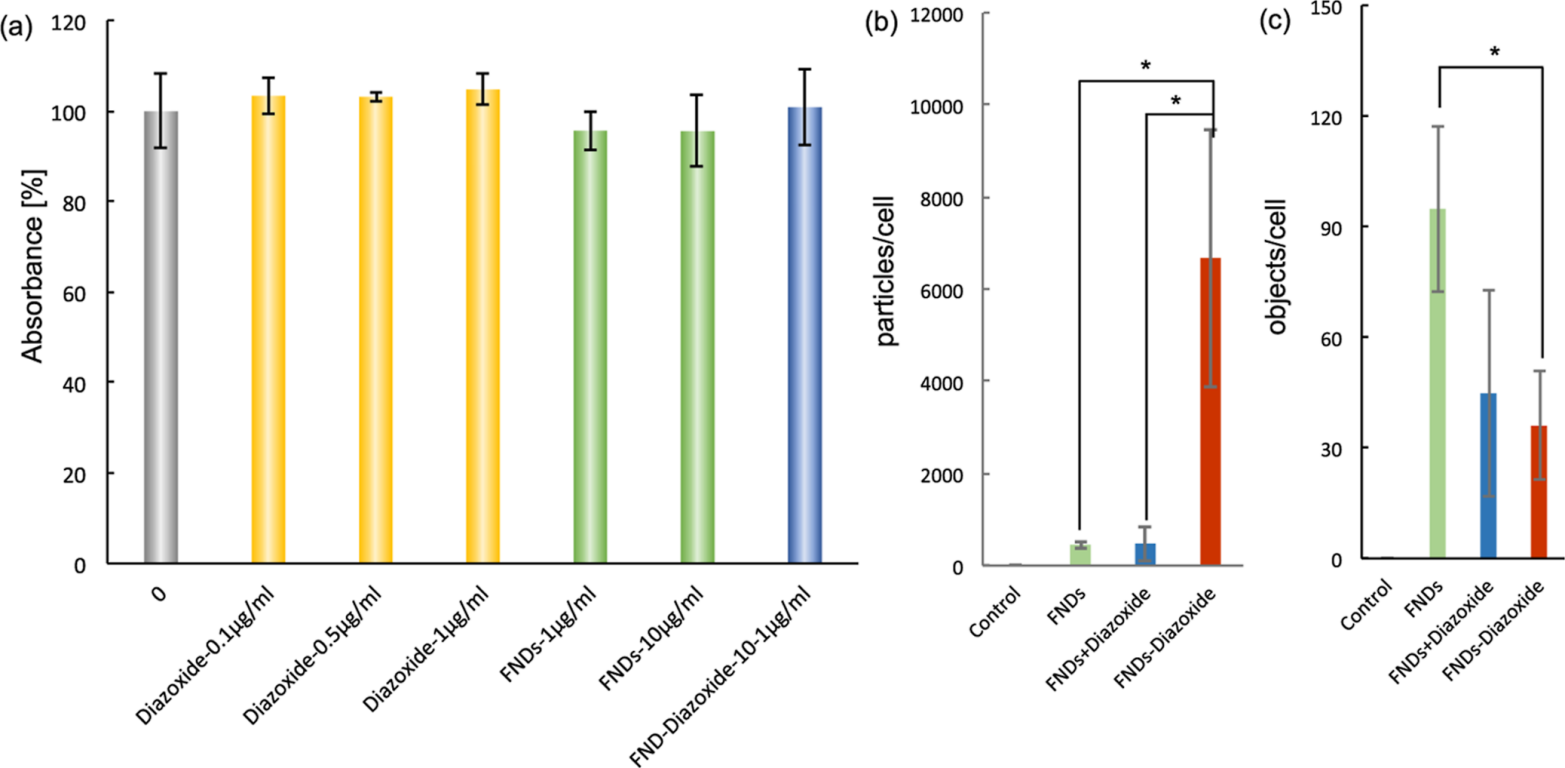
FND uptake and biocompatibility. (a) Metabolic activity determined by the XTT assay. There are no significant differences between any of the tested groups, indicating excellent biocompatibility. Data represent the mean ± SD (*n* = 5), (b,c) show the quantification of particles and objects within cells. Data represent the mean ± SD (*n* = 5, *P < 0.05). Experiments were repeated 3 times.

HeLa cells were incubated with FNDs and FND–diazoxide for 12 h, and the LSCM imaging results are shown in Figure 4. The cytoskeleton was stained with FITC-phalloidin (FP) (EX 488 nm, EM 499–552 nm) and is shown in green. FNDs and FND–diazoxide (EX 561 nm, EM 645–758 nm) are shown in red, and the nuclei are stained with DAPI (EX 405 nm, EM 424–485 nm) and shown in blue. The LSCM images show that diazoxide-modified FNDs are internalized in higher numbers in Hela cells than unmodified FNDs. Further analysis of the images was used to quantify FND uptake of each cell. Figure 5b shows that the number of particles in FND–diazoxide-treated cells is 1500 times higher (*P* < 0.01) than for the FND group. Taken together, the above results indicate that diazoxide modification significantly improves the delivery of FNDs into HeLa cells. There is also a clear difference between the number of objects and particles, which indicates that particles tend to form aggregates once they are inside cells.

### 3.4 Biocompatibility in HeLa Cells

The effect of diazoxide, FNDs, and FND–diazoxide on the viability of HeLa cells was investigated after 24 h of incubation (Figure 5a). The cell’s metabolic activity, a good indicator of cell viability, is not significantly different from the control for any of the tested groups. This confirms the excellent biocompatibility of FNDs that is known from the literature.^1,2^ Besides, the conjugation with diazoxide also does not affect viability.

### 3.5 Total ROS Production

**Figure 6:**
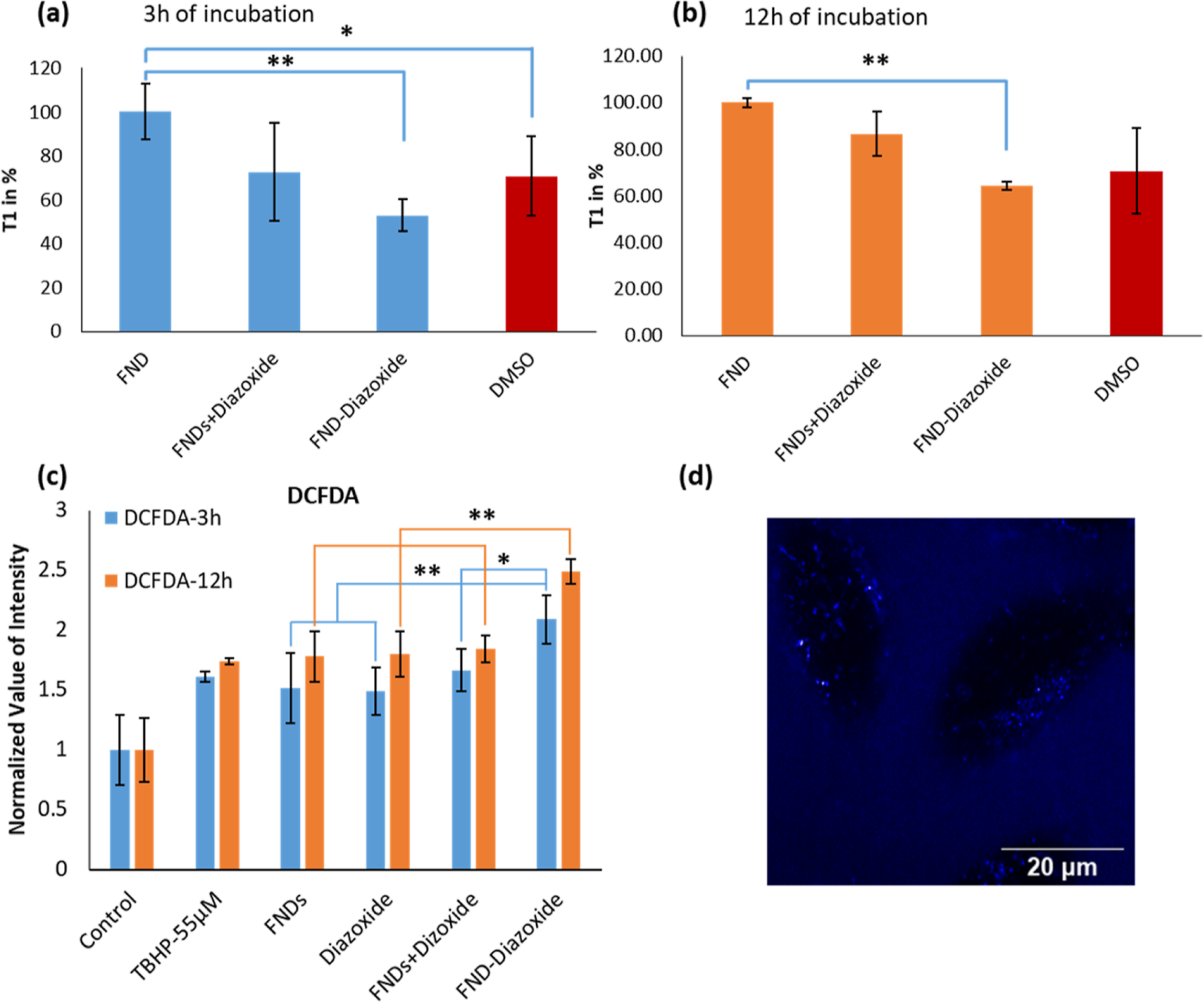
Free radical detection results. (a,b) T1 relaxation time of FNDs in % for the mixture of FNDs and diazoxide and FND–diazoxide after a 3 or 12 h incubation in living HeLa cells. For each experiment, the diazoxide concentration was 1 μg/mL. The decay in the presence of diazoxide is faster, both when it is linked to FNDs or added separately. Data represent the mean ± SD (*n* = 4,*P < 0.05, **P < 0.01). (c) The same groups were tested for ROS production. This is measured by observing the conversion of DCFDA to DCF. The more ROS and thus DCF there is, the higher the fluorescent signal a sample emits. Data represent the mean ± SD (**P < 0.01). Each experiment was repeated with four different FNDs in four independent samples. (d) HeLa cells under our homemade T1 detection system. The image shows the fluorescence intensity detected in red (above 600 nm).

To evaluate the total ROS production inside HeLa cells, we used a DCFDA assay inside cells. To this end, HeLa cells were treated with either PBS, 10 μg/mL FNDs, a mixture of 10 μg/mL FNDs, and 1 μg/mL diazoxide, or FND–diazoxide (including 10 μg/mL FNDs and 1 μg/mL diazoxide) for 3 or 12 h. Figure 6c shows the DCFDA analysis of HeLa cells, including FNDs, a mixture of FNDs and diazoxide (FND + diazoxide), and diazoxide-modified FNDs (FND–diazoxide). After a 3 or 12 h incubation, there is no significant difference between the groups except for the FND–diazoxide group. Figure 6c shows that the fluorescent intensity and thus ROS production of FND–diazoxide-treated groups are much stronger than other groups. For a 3 h incubation, the values are 47.9% for the FND + diazoxide group (*P* < 0.01), 60.4% for the diazoxide group (*P* < 0.01), 43.5% for the FND group (*P* < 0.05), and 117.4% for the control group (*P* < 0.01). For a 12 h incubation, the values are 35.3% for the FND + diazoxide group (*P* < 0.01), 38.2% for the diazoxide group (*P* < 0.05), 39.9% for the FND group (*P* < 0.05), and 148.7% for the control group (*P* < 0.01). The increasing fluorescence intensity of the FND–diazoxide group suggests a possible effect of FND–diazoxide causing more accumulative ROS production. Furthermore, according to cell uptake results, the increasing ROS production can come from more FND–diazoxide inside cells.

### 3.6 T1 Relaxation Time Analysis

As shown in Figure 6a,b, the T1 relaxation time for the FNDs with diazoxide is faster than the T1 of FNDs only. This means that there are more radicals generated in the presence of diazoxide inside the cell. This is in good agreement with the literature,^69^ where it was found that adding diazoxide to the cell can stimulate the opening of K(ATP) channels, thus increasing the generation of ROS. However, there are a few distinct differences between the literature and our measurements. The values in the literature are typically from ensembles of cells, while we obtain single cell information with subcellular resolution. While conventional assays provide the history of the sample, we obtain the current stage. Finally, the data from conventional assays is dominated by more abundant nonradical species of ROS, while we are specific for radicals. Moreover, we observed that the T1 relaxation time for the FND–diazoxide is shorter than T1 of the mixture of FNDs and diazoxide after incubating with Hela cells for 3 or 12 h. FNDs linked to the diazoxide can detect a magnetic change more effectively compared to the mixture of NDs and diazoxide since FNDs are closer to the diazoxide in FND–diazoxide group.

**Figure 7:**
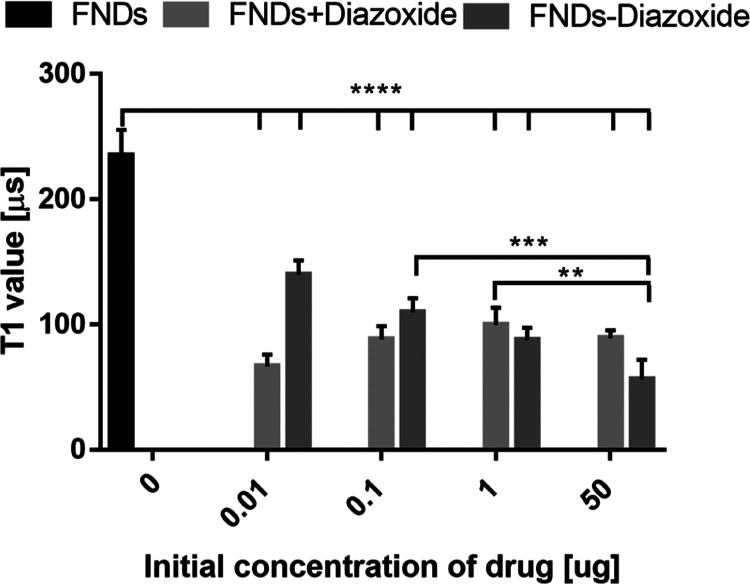
Concentration dependency of T1. We loaded the FNDs with different concentrations of drug in the FND + diazoxide and the FND–diazoxide groups. We then detected the free radical response by the cells after 3 h. For each condition, we measured five particles and show the average. The error bars represent standard deviations. Statistical significance is indicated by **P < 0.01, ***P < 0.0002, ***P < 0.0001.

Next, we used different concentrations of the diazoxide derivate to determine if there is a dose response (see Figure 7). In the FND + diazoxide group, we observed a relatively large decrease in T1 for all concentrations. This is likely due to the fact that in this group, the drug is available in a relatively high concentration early on in the experiment throughout the cell. Also, in the FND–diazoxide group, we observed a decrease in T1 in all the concentrations. Interestingly, in this case, we observed a concentration dependency. In the FND–diazoxide group, overall less drug is released at this time point, but it is released at the location of the particle.

## 4 Conclusions

Here, we have demonstrated that FND–diazoxide is able to deliver diazoxide derivatives into HeLa cells. We could demonstrate that these particles show sustained release compared to free drugs. Furthermore, our approach allows to track the particles and measure the local response of the drug directly at the location of the particle and where the drug is released. The free radical response is also concentration-dependent in the FND–diazoxide group. Detection was achieved by making use of the quantum sensing abilities of FNDs. Since FNDs do not bleach, it is possible to follow single particles in single cells over the course of the release experiment. This unique ability offers a powerful tool to deliver drugs and measure their impact on cells locally.

## Supporting Information Available

DLS Characterization of FND–diazoxide and FNDs and calibration for determining the amount of free diazoxide by HPLC (PDF)
