# Supplementary material for: Functionalized Fluorescent Nanodiamonds for Simultaneous Drug Delivery and Quantum Sensing in HeLa Cells
Source: ACS Appl Mater Interfaces. 2022 Aug 19;14(34):39265–73. doi: 10.1021/acsami.2c11688 (PMC9437893; doi:10.1021/acsami.2c11688)
Supplement: Supplementary file 1 — am2c11688_si_001.pdf [file am2c11688_si_001.pdf]

Supplementary information

Functionalized Fluorescent Nano Diamonds for simultaneous drug delivery and quantum-sensing in HeLa cells

Yuchen Tian[a$], Anggrek C. Nusantara[a,$], Thamir Hamoh[a,], Aldona Mzyk[a], Xiaobo Tian[b], Felipe Perona Martinez[a], Runrun Li[a], Hjalmar P. Permentier[b], Romana Schirhagl[a,*]

a Department of Biomedical Engineering, Groningen University, University Medical Center Groningen, Antonius Deusinglaan 1, 9713 AW Groningen, Netherlands, romana.schirhagl@gmail.com

b Department of Analytical Biochemistry, Interfaculty Mass Spectrometry Center, Groningen Research Institute of Pharmacy, University of Groningen, A. Deusinglaan 1, Groningen 9713 AV, The Netherlands

*Correspondence: romana.schirhagl@gmail.com

$ These authors contributed equally to this work.

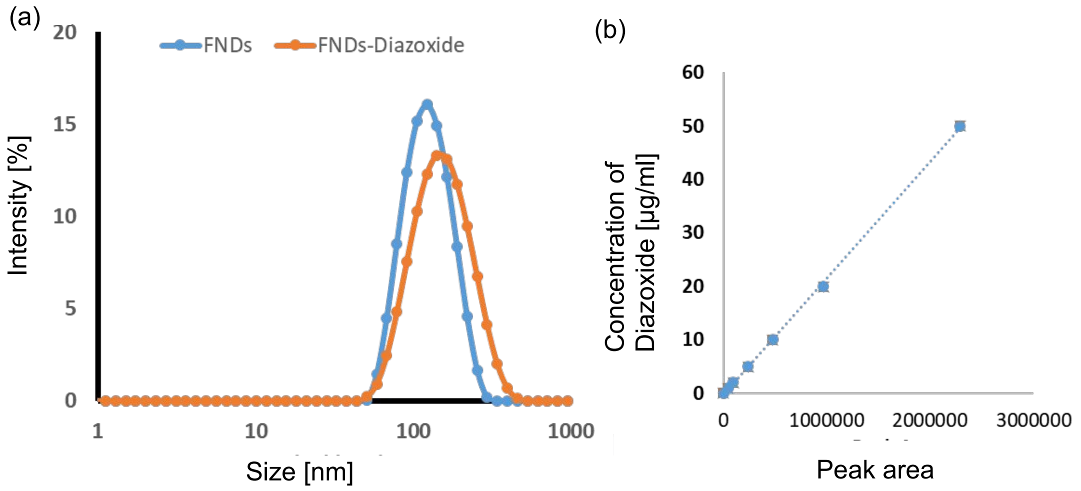

Fig. S1. Characterization of FNDs-Diazoxide, a) size distribution of FNDs, and FNDs-Diazoxide. b) Calibration for determining the amount of free Diazoxide by HPLC
